# Complement System in Brain Architecture and Neurodevelopmental Disorders

**DOI:** 10.3389/fnins.2020.00023

**Published:** 2020-02-05

**Authors:** Juliana Magdalon, Fernanda Mansur, André Luiz Teles e Silva, Vitor Abreu de Goes, Orly Reiner, Andréa Laurato Sertié

**Affiliations:** ^1^Center for Experimental Research, Hospital Israelita Albert Einstein, São Paulo, Brazil; ^2^School of Medicine, Faculdade Israelita de Ciências da Saúde Albert Einstein, São Paulo, Brazil; ^3^Department of Genetics and Evolutionary Biology, University of São Paulo, São Paulo, Brazil; ^4^Department of Molecular Genetics, Weizmann Institute of Science, Rehovot, Israel

**Keywords:** complement system, neural progenitor proliferation, neurogenesis, neuronal migration, synapse refinement, neurodevelopmental disorders

## Abstract

Current evidence indicates that certain immune molecules such as components of the complement system are directly involved in neurobiological processes related to brain development, including neurogenesis, neuronal migration, synaptic remodeling, and response to prenatal or early postnatal brain insults. Consequently, complement system dysfunction has been increasingly implicated in disorders of neurodevelopmental origin, such as schizophrenia, autism spectrum disorder (ASD) and Rett syndrome. However, the mechanistic evidence for a causal relationship between impaired complement regulation and these disorders varies depending on the disease involved. Also, it is still unclear to what extent altered complement expression plays a role in these disorders through inflammation-independent or -dependent mechanisms. Furthermore, pathogenic mutations in specific complement components have been implicated in the etiology of 3MC syndrome, a rare autosomal recessive developmental disorder. The aims of this review are to discuss the current knowledge on the roles of the complement system in sculpting brain architecture and function during normal development as well as after specific inflammatory insults, such as maternal immune activation (MIA) during pregnancy, and to evaluate the existing evidence associating aberrant complement with developmental brain disorders.

## Introduction

The complement system has been increasingly implicated in multiple physiological and homeostatic functions, including development and maintenance of the central nervous system (CNS) (Orsini et al., 2014; Coulthard et al., 2018b), in addition to its well documented roles in immune surveillance and host defense against pathogens and injured cells (Kolev et al., 2014). Virtually all complement components can be locally produced in the brain, where they play critical roles in almost every aspect of normal brain development, including neurogenesis (Rahpeymai et al., 2006; Coulthard et al., 2017; Gorelik et al., 2017a, b), neuronal migration (Gorelik et al., 2017a, b), and synaptic refinement (Schafer et al., 2012; Stephan et al., 2012). Moreover, the complement system plays an important role in the maintenance of uninjured brain homeostasis, protecting from infection and inflammation, eliminating damaged cells and supporting regeneration (Alawieh et al., 2015; Hammad et al., 2018). However, in the injured, aged or diseased CNS, the synthesis of components of the complement pathway markedly increases and contributes to local inflammation and tissue damage, which may lead to blood brain barrier injury (Orsini et al., 2014; Hammad et al., 2018). Consequently, the brain parenchyma can be invaded by a number of peripheral blood-derived inflammatory cells and molecules, including complement proteins, which amplify local damage and brain malfunction (Brennan et al., 2012).

Given the multi-faceted functions of complement in CNS development, dysfunction of specific components of the complement system has been increasingly linked to developmental brain disorders, including 3MC syndrome, a rare autosomal recessive disorder with facial dysmorphism, growth deficiency and cognitive deficit (Sirmaci et al., 2010; Rooryck et al., 2011; Munye et al., 2017), as well as to more prevalent and genetically complex disorders, such as schizophrenia (Sekar et al., 2016) and autism spectrum disorder (ASD) (Warren et al., 1991; Fagan et al., 2017). However, despite recent advances, several aspects of the involvement of the complement system in the pathogenesis of these complex neurodevelopmental disorders are still unclear. It is not yet fully established, for example, whether aberrant complement expression produced locally during brain development plays an etiological role in these disorders independently of any role in inflammation, or whether aberrant complement activation both systemically and in the CNS, as a result of inflammatory insults during prenatal or early postnatal neurodevelopment, also plays a part in the pathophysiology of these disorders as a secondary event.

In support of the first possibility are, as abovementioned, recent findings reporting multiple emerging novel non-inflammatory roles of complement in every stage of brain development (Coulthard et al., 2018b), as well as data from genetic studies showing an association between risk variants in complement genes and neurodevelopmental disorders (Warren et al., 1991; Odell et al., 2005; Sekar et al., 2016). In addition, there are studies suggesting that the neuropathological and behavioral phenotypes in genetically modified mouse models with aberrant complement expression in the brain parallel known features of these human developmental brain disorders (Chu et al., 2010; Perez-Alcazar et al., 2014; Sekar et al., 2016; Comer et al., 2019).

In support of the latter possibility are data from different studies describing the presence of activated astrocytes and microglia in brains as well as the presence of altered expression of immune molecules, including complement components, in peripheral blood and/or cerebrospinal fluid obtained from individuals with schizophrenia and ASD (Mayilyan et al., 2006; Corbett et al., 2007; Morgan et al., 2010; Ashwood et al., 2011; Ishii et al., 2018). In addition, recent studies using animal models have suggested a role for the complement system in brain and behavioral abnormalities in offspring associated with prenatal maternal immune activation (MIA) (Pedroni et al., 2014; McDonald et al., 2015). In humans, severe maternal infections during pregnancy have been highlighted as a potential risk factor for these neurodevelopmental disorders (Patterson, 2009, 2011; Malkova et al., 2012; Knuesel et al., 2014; Coiro et al., 2015). One possibility is that immune molecules released by the maternal immune response can cross the placenta and enter the fetal brain, where they contribute to the pathological and behavioral changes. The ongoing immune dysregulation in the brain and the peripheral immune system of individuals with these neurodevelopmental diseases suggest that MIA or other inflammatory insults during prenatal or early postnatal development may induce an irregular immune phenotype that persists into adulthood.

This review aims to highlight the importance of the complement system in regulating the development of the healthy and diseased brain. First, we provide an overview of the well-known concepts of complement system activation in the immune system context. Then, we discuss recent progress in understanding the roles of the complement system in important physiological processes of normal brain development, as well as initial findings suggesting a potential role for complement in neuropathological and behavioral abnormalities in MIA offspring. Finally, we evaluate the current evidence for the involvement of the complement system dysfunction in disorders that trace their origin to abnormal brain development, including schizophrenia, ASD, Rett syndrome, and 3MC syndrome.

## Complement System Activation: a Background

**FIGURE 1:**
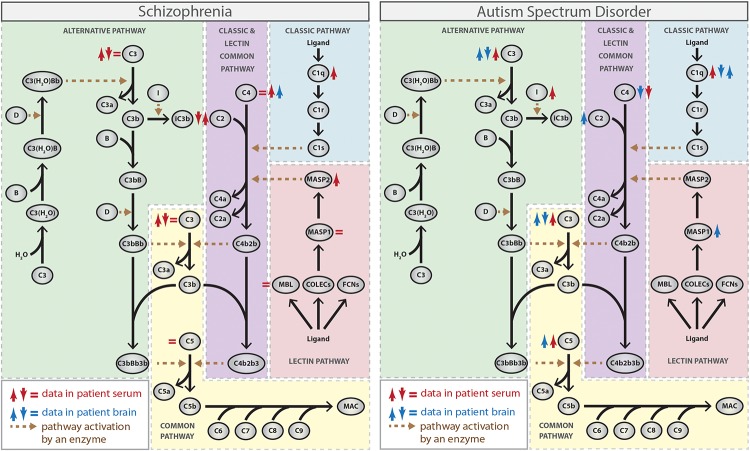
Complement activation pathways and the activity or expression of their individual components (either protein or mRNA) in schizophrenia and autism spectrum disorder (ASD). ↑ red, increased protein activity or expression in blood from patients; ↓ red, decreased protein activity or expression in blood from patients; ↑ blue, increased RNA expression in brain tissues from patients; ↓ blue, decreased RNA expression in brain tissues from patients (Spivak et al., 1989, 1993; Warren et al., 1994; Wong et al., 1996; Maes et al., 1997; Shcherbakova et al., 1999; Hakobyan et al., 2005; Mayilyan et al., 2006; Corbett et al., 2007; Boyajyan et al., 2010; Momeni et al., 2012; Nardone et al., 2014; Li et al., 2016; Sekar et al., 2016; Fagan et al., 2017; Shen et al., 2018). The Figure does not distinguish between strong and weak evidence.

The complement cascade is composed of several soluble and membrane-bound proteins that are mainly secreted by the liver, but also by leukocytes, adipocytes, cells in the CNS (such as neurons, astrocytes, and microglia), among others (Veerhuis et al., 2011; Bajic et al., 2015). Complement is activated through the classical, lectin and alternative pathways, which are initiated by different stimuli and result in the generation of: (1) opsonins (such as C3b and C4b), which recognize and bind to target cells to facilitate their removal by phagocytic cells that express complement receptors (such as complement receptor type 1, CR1); (2) anaphylatoxin proteins (such as C3a and C5a), which are proinflammatory peptides that interact with and activate immune cells through interaction with their receptors (C3a receptor, C3aR, and C5a receptor, C5aR); (3) terminal membrane attack complex (MAC), which are pores that disrupt lipid bilayers and lyses targeted (opsonized) pathogens or self-damaged cells (Ricklin et al., 2010; Bajic et al., 2015; Figure 1).

The classical pathway is initiated by C1 complex activation, which consists of a recognition molecule C1q and two copies of each of the homologous C1r and C1s serine proteases. Initially, C1q binds to one of its ligands, such as antibodies, C-reactive protein and some structures on invading microorganisms or apoptotic cells, which triggers a conformational change within the C1 complex resulting in the activation of C1r, which subsequently cleaves and activates C1s (Gaboriaud et al., 2004). The activated C1s then cleaves the complement protein C4 into the fragments C4a and C4b. C4b may then opsonize the activator and facilitates phagocytosis. Additionally, C4b binds to the complement protein C2, which is subsequently cleaved by C1s into the fragments C2a and C2b. C2a remains attached to C4b and forms the C3 convertase C4b2b (previously termed C4b2a) of the classical pathway, which cleaves the complement protein C3 into the fragments C3a and C3b, triggering the final common part of the complement cascade (Muller-Eberhard et al., 1967; Dunkelberger and Song, 2010; Figure 1).

The lectin pathway is functionally similar to the classical pathway and also leads to formation of the C3 convertase C4b2b. However, the lectin pathway is initiated by binding of collectins [mannose-binding lectin (MBL), collectin-10 (COLEC10), and collectin-11 (COLEC11)] or ficolins (ficolin-1, ficolin-2, and ficolin-3) to sugar moieties or certain acetyl groups present on the surface of a large variety of pathogens, which leads to activation of the MBL-associated serine proteases (MASP) 1 and 2, structurally and functionally similar to C1r and C1s (Garred et al., 2016). While both enzymes cleave C2, only MASP2 cleaves C4, generating the C3 convertase C4b2b in a reaction analogous to the classical pathway (Chen and Wallis, 2004; Heja et al., 2012; Figure 1).

In contrast to the other two pathways, the alternative pathway does not depend on the recognition of exogenous materials and is constitutively active at low levels through a process called “tickover.” This pathway is activated by spontaneous hydrolysis of plasma C3 to form C3(H_2_O), which binds to factor B. Factor D cleaves factor B to form Ba and Bb, which then generates C3(H_2_O)Bb, the initial alternative pathway C3 convertase that can cleave C3 into C3a and C3b (Bexborn et al., 2008; Lachmann, 2018). Subsequently, C3b generated from any of the pathways can bind to factor B, which is cleaved by factor D, generating C3bBb, the main C3 convertase of the alternative pathway. C3bBb produces more C3b molecules, promoting an amplification loop for the entire system. Also, factor I cleaves C3b to iC3b, allowing iC3b to interact with leukocyte complement receptors (CR3 and CR4) and trigger the inflammatory response (Lachmann, 2009; Figure 1).

Therefore, the terminal phase of the complement cascade is similar for the classical, lectin, and alternative pathways and leads to the generation of C3 convertases. The incorporation of C3b into the C3 convertases generates C5 convertases (C4b2b3b for the classical and lectin pathways, and C3bBbC3b for the alternative pathway) that cleave C5 into C5a, which binds to its receptors on phagocytic cells, and C5b, which associates sequentially to other complement proteins (C6, C7, C8, and C9) forming the MAC and leading to cell lysis (Muller-Eberhard, 1985; Bajic et al., 2015; Figure 1).

It should be noted that the activity of the complement system is tightly regulated to protect host cells from indiscriminate attack and limit the deposition of complement molecules on pathogen and host surfaces. Among the complement regulators is SERPING1 (or C1-inhibitor), which binds to and inactivates C1r, C1s, and MASP-1/2 proteases, thus leading to inhibition of the classical and lectin pathways (Ricklin et al., 2010). Other regulators include: factor H, which acts as a cofactor for complement factor I (CFI) in the C3b cleavage and also favors the dissociation of factor Bb from C3b (Lachmann, 2009); CSMD1, which acts as a cofactor to CFI-mediated degradation of C4b and C3b and also inhibits MAC assembly by preventing binding of C7 to the C5b-6 complex (Escudero-Esparza et al., 2013); and CD59, which inhibits MAC formation by preventing binding of C9 to the C5b-8 complex (Farkas et al., 2002).

## Complement System in the Development of Healthy and Diseased Brain

### Complement System in Neurogenesis

Besides playing a critical role during embryogenesis, the generation of new neurons is sustained throughout adulthood in the mammalian brain through the proliferation and differentiation of neural progenitor cells (NPC) present in neurogenic niches, mainly the subventricular zone of lateral ventricles and the subgranular zone of the hippocampal dentate gyrus (Fuentealba et al., 2012). Studies using animal and *in vitro* cell models have shown important roles for specific complement components in the regulation of neurogenesis both in the embryonic and adult brain under normal physiological conditions.

It has recently been shown that mouse embryos deficient for *C3*, *Masp2* or treated with C3aR antagonist exhibit increased proliferation of NPC in the brain ventricular or subventricular zones, suggesting that these complement components inhibit NPC proliferation at early stages of cortical development (Gorelik et al., 2017a; Coulthard et al., 2018a). It is noteworthy, however, that an opposite trend was observed for *C3aR* knockout mouse embryos, that display decreased proliferation of NPC within the ventricular zone (Coulthard et al., 2018a; Table 1). This discrepancy between the use of C3aR pharmacological blocker and *C3aR* knockout may be attributed in part to combinatorial modulation of other signaling pathways in the absence of C3aR during the entire developmental period (Coulthard et al., 2018a). In the context of adult mouse brain, previous studies have shown that young adult mice lacking *C3*, *C3aR* or treated with C3aR antagonist exhibit reduced neurogenesis from NPC in the neurogenic niches, possibly due to impaired NPC differentiation rather than decreased proliferation of these cells (Rahpeymai et al., 2006). These findings were further corroborated by an *in vitro* study using NPC isolated from adult mouse brain showing that C3a stimulates their neuronal differentiation without altering their survival and proliferation (Shinjyo et al., 2009; Table 1). Consistent with the findings that C3a/C3aR signaling regulates neurogenesis, adult *C3aR* knockout mice show deficits in memory (Coulthard et al., 2018a).

**TABLE 1:** Summary of the phenotypes observed after disturbances in the expression of individual components of the complement pathway.

**Functional alteration**	**Pathway**	**Model**	**NPC proliferation**	**NPC differentiation**	**Neuronal migration**	**Brain wiring**	**References**
*C1q* knockout	Cl	Mouse embryo			=		Gorelik et al., 2017a
		Postnatal mouse				↑	Comer et al., 2019, Bialas and Stevens, 2013
*C1s* knockdown	Cl	Mouse embryo			↓		Gorelik et al., 2017a
*Masp1* knockout	L	Mouse embryo			↓		Gorelik et al., 2017a
*Masp1* knockdown	L	Mouse embryo	=		↓		Gorelik et al., 2017a
		Zebrafish embryo			↓*		Rooryck et al., 2011
*Masp2* knockout	L	Mouse embryo			↓		Gorelik et al., 2017a
*Masp2* knockdown	L	Mouse embryo	↑		↓		Gorelik et al., 2017a
*Colec11* knockdown	L	Zebrafish embryo			↓*		Rooryck et al., 2011
*C4* knockout	Cl and L	Postnatal mouse				↑	Sekar et al., 2016
*C4* overexpression	Cl and L	Postnatal mouse				↓	Perez-Alcazar et al., 2014
*C3* knockout	C	Mouse embryo	↑		↓		Gorelik et al., 2017a
		Postnatal mouse				↑	Schafer et al., 2012; Bialas and Stevens, 2013
		Adult mouse		↓			Rahpeymai et al., 2006
*C3* knockdown	C	Mouse embryo	↑		↓		Gorelik et al., 2017a
C3a antibody	C	*Xenopus* embryo			↓*		Gorelik et al., 2018
		*Xenopus* NCC *in vitro*			↓*		Gorelik et al., 2018
*C3aR* knockout	C	Mouse embryo	↓				Shinjyo et al., 2009
		Adult mouse		↓			Rahpeymai et al., 2006
*C3aR* knockdown	C	*Xenopus* embryo			↓*		Gorelik et al., 2018
		*Xenopus* NCC *in vitro*			↓*		Gorelik et al., 2018
C3aR antagonist	C	Mouse embryo	↑				Shinjyo et al., 2009
		Adult mouse		↓			Rahpeymai et al., 2006
		Postnatal rat granule cell *in vitro*			=		Shinjyo et al., 2009
C3aR agonist	C	Mouse embryo	↓				Shinjyo et al., 2009
		Mouse embryo NPC *in vitro*	↓				Shinjyo et al., 2009
		Postnatal rat granule cell *in vitro*	=		↑		Shinjyo et al., 2009
		Adult mouse NPC *in vitro*		↑	=		Benard et al., 2008
*CR3* knockout	C	Postnatal mouse				↑	Schafer et al., 2012
*C5aR* knockout	C	Adult mouse		**=**			Marin et al., 2010
C5aR antagonist	C	Mouse embryo	↓				Coulthard et al., 2017
		Postnatal rat cerebellum	=				Shinjyo et al., 2009
		Postnatal rat granule cell *in vitro*	=				Shinjyo et al., 2009
C5aR agonist	C	Mouse embryo	↑				Coulthard et al., 2017
		Human and mouse NPC *in vitro*	↑				Coulthard et al., 2017
		Postnatal rat cerebellum	↑				Shinjyo et al., 2009
		Postnatal rat granule cell *in vitro*	↑		=		Shinjyo et al., 2009
		Adult mouse NPC *in vitro*		=	=		Benard et al., 2008
*Serping1* knockout	Ci	Mouse embryo	↓		↓		Gorelik et al., 2017b
*Serping1* knockdown	Ci	Mouse embryo	↓		↓		Gorelik et al., 2017b

Cl, Classical pathway; L, Lectin pathway; C, Common final pathway; Ci, Complement inhibitor;=, No difference; *, Neural crest cells.

Constitutive deficiency of *C5aR* and acute pharmacological blockade of C5aR during neurogenesis also caused opposing phenotypes of NPC proliferation. While the use of C5aR antagonist inhibits NPC proliferation in the ventricular zone of mouse embryos and lead to brain microstructural alterations and behavioral deficits (such as heightened anxiety, impaired coordination, and short-term memory) later in life (Coulthard et al., 2017), *C5aR* knockout mice exhibit increased proliferation of NPC within the ventricular zone (Coulthard et al., 2018a; Table 1). In addition, while in the postnatal rat cerebellar cortex a C5aR agonist was shown to stimulate proliferation of immature granule neurons, which suggests a role for the C5a-C5aR axis in the cerebellar histogenesis (Benard et al., 2008), C5a-C5aR1 signaling seems not to be involved in NPC proliferation and differentiation in the neurogenic niches of the adult brain (Bogestal et al., 2007; Shinjyo et al., 2009; Table 1).

Interestingly, it has recently been shown that mouse embryos deficient in the *Serping1* gene, a known inhibitor of the classical and lectin pathways of the complement system, display decreased proliferation of both ventricular zone (radial) and intermediate (basal) progenitors during development of the cortex, suggesting that SERPING1 stimulates proliferation of NPC at early stages of cortical development (Gorelik et al., 2017b; Table 1). However, it is still unknown whether this function of SERPING1 is either dependent or independent on downstream activation of the complement system.

Together, the abovementioned studies suggest a role mostly for the anaphylatoxins in NPC proliferation and differentiation in the absence of other factors of the canonical pathogen-initiated complement activation routes. Also, these studies suggest that the spatiotemporal expression pattern of these complement components in different subsets of NPC seems to determine their role in progenitor neurogenesis.

### Complement System in Neuronal Migration

Neuronal migration is an essential phenomenon for proper brain formation and establishment of neural circuit since most neurons must move from their birth position to their final location in the brain. During development, excitatory neurons arising from the proliferative neuroepithelium surface (the ventricular zone) exhibit mainly radial migration, in which early postmitotic neurons migrate along the processes of radial glial progenitors to their correct laminar position within the cortical plate. Inhibitory neurons are born in the ganglionic eminences and exhibit, initially, tangential migration, in which nascent neurons move in trajectories that are parallel to the ventricular surface (Marin et al., 2010). There is mounting evidence showing that the complement system plays an important role in the radial migration of pyramidal neurons during normal brain development.

A recent study has uncovered a direct role for the lectin arm of the complement system in radial neuronal migration in the developing cerebral cortex. *C3*-, *Masp1-* or *Masp2*-deficient mice exhibit impairments in radial migration resulting in improper positioning of neurons and disorganized cortical layers (Gorelik et al., 2017a). Importantly, the migration deficits observed in *C3-* or *Masp2*-deficient mice were partially rescued by addition of polypeptides that mimic C3 cleavage products, C3aR agonist or a dual C3aR/C5aR agonist, suggesting that activation of the lectin cascade leading to C3 cleavage, C3a and C5a generation and activation of both C3aR and C5aR are necessary for proper radial neuronal migration and cortical development (Gorelik et al., 2017a; Table 1).

It is also noteworthy that components of the lectin complement pathway, including MASP1 and CL-K1 (encoded by the *Colec11* gene) proteins, as well as the C3a-C3aR axis, were shown to behave as early guidance cues to direct the migration of neural crest cells during embryonic vertebrate development, since deficiency of these components in zebrafish or *Xenopus* causes craniofacial abnormalities (Rooryck et al., 2011) or disorganized collective cell migration (Carmona-Fontaine et al., 2011; Table 1).

Abnormal radial neuronal migration was also observed in *Serping1* knockout and knockdown mice but, unexpectedly, deficiency of SERPING1 resulted in a small but significant decrease in C3b levels, suggesting that the complement pathways are not being activated (Gorelik et al., 2017b). Moreover, addition of C3 mimicry cleavage products, or addition of a dual C3aR/C5aR agonist, but not a C3a peptide or a specific C3aR agonist, significantly improved impaired neuronal migration (Table 1). These findings corroborate a role for C3 cleavage and downstream complement activation at the level of C5a in controlling normal migration of newly born neurons to the cortical plate (Gorelik et al., 2017b). Interestingly, it has been recently shown that *C3* knockout mouse embryos display reduced activity of the small GTPase Rac1 and reduced phosphorylation of Cofilin, a cytoskeleton protein, in migrating neurons entering the cortical plate. This suggests that Rac1 may be one of the key downstream mediators of the complement activity to control cytoskeletal remodeling required for proper neuronal migration in the developing brain (Gorelik et al., 2018).

In agreement with the results obtained with mouse embryos, previous studies have shown that adult mice deficient for C3a/C3aR signaling show impaired migration of both neuroblasts and newly formed neurons from the brain neurogenic niches (Rahpeymai et al., 2006), and that C3a stimulates migration of *in vitro* mouse adult brain NPC in response to low concentrations of the chemokine stromal cell-derived factor 1 alpha (Shinjyo et al., 2009). Also, the C3a-C3aR signaling was shown to regulate migration of immature granule neurons in the postnatal rat cerebellar cortex, suggesting that this signaling also plays a role in the cerebellum ontogenesis (Benard et al., 2008; Table 1).

Taken together, the existing findings indicate that functional activation of the lectin complement pathway and consequent production of anaphylatoxins are important for proper neuronal migration and correct positioning of neurons during brain development.

### Complement System in Brain Wiring

Formation of precise neural circuitry during development is essential for proper functions of the CNS. Synaptic contacts are generated in excess during early phases of development, and postnatally unnecessary synapses are eliminated while functionally important synapses are strengthened to construct appropriate neural circuits (Rakic et al., 1986; Riccomagno and Kolodkin, 2015). Much of the current understanding about the mechanisms underlying synapse refinement during CNS development has been studied using the mouse retinogeniculate pathway. Early in development, axons from retinal ganglion cells form transient connections with neurons of dorsal lateral geniculate nucleolus (dLGN) of the thalamus and, during early post-natal development, these connections are sculpted through the pruning of redundant synapses (Hooks and Chen, 2006). Important studies have revealed that mice deficient for complement *C1q*, *C3*, *C4* or microglia-specific *CR3* exhibit impaired elimination of retinogeniculate synapses and defects in eye-specific segregation (Stevens et al., 2007; Schafer et al., 2012; Sekar et al., 2016), suggesting that the classical complement system plays a crucial role in synapse pruning during development (Table 1). Mechanistically, it has been proposed that transforming growth factor (TGF)-β released by retinal astrocytes induces the expression of C1q in retinal ganglion cells, which is transported from cell bodies along axons to the dLGN, where it is released to bind weak synapses (Bialas and Stevens, 2013). Similar to the immune system, the binding of C1q and formation of C1 complex results in activation of the classical complement pathway, cleavage of C4, formation of C3 convertase and then production of C3a and C3b/iC3b fragments. Activated C3 (iC3b) binds to CR3 in microglia ultimately promoting the engulfment of overlapping and weaker synapses (Schafer et al., 2012).

It is noteworthy that, in accordance with the abovementioned data using the mouse retinogeniculate pathway, *C1q-* or *C3-*deficient mice exhibit increased number of excitatory synapses in the cortex and hippocampus, respectively (Chu et al., 2010; Perez-Alcazar et al., 2014), epilepsy (Chu et al., 2010) or abnormal hippocampus-dependent learning (Perez-Alcazar et al., 2014; Table 1). Also, *C3* knockdown specifically in the prefrontal cortex lead to repetitive behavior and impaired social interaction in mice, possibly due to reduced synaptic pruning (Fagan et al., 2017). In addition, a study using cortical wild type neurons co-cultured with astrocytes derived from *IkBα* knockout mice, which overexpress the transcription factor NFkB and consequently complement C3, has shown that C3 released by astrocytes acts through neuronal C3aR reducing excitatory synaptic density and dendritic length and complexity (Lian et al., 2015). Moreover, it was shown that overexpression of *C4* in mouse prefrontal cortex leads to dendritic spine dysgenesis, reduced connectivity in cortical neurons, enhanced microglia-mediated engulfment of synapses and deficits in social interactions (Comer et al., 2019; Table 1).

Although the mechanisms that drives the opsonization of weaker synapses are not yet fully understood, a recent study using whole cortical tissue from newborn and adult mice has shown that C1q is predominantly localized to the presynaptic region of the labeled synapses, and that C1q-tagged synapses display downregulation of proteins associated with synaptic transmission and increased expression of apoptotic markers, suggesting that weaker synapses induce apoptotic-like mechanisms that attracts C1q and then triggers synaptic elimination by microglia (Gyorffy et al., 2018).

Altogether, the findings abovementioned suggest that microglia- and astroglia-mediated complement-dependent synaptic refinement occurs in different regions of the developing CNS, and that alterations in this process affect behavior in animal models.

### Complement System in Brain Pathology Associated With Prenatal Maternal Immune Activation

The developing brain is particularly vulnerable to environmental insults, such as ischemic and inflammatory insults, that can cause injury and long-term neurodevelopmental abnormalities manifesting as cognitive difficulties or behavioral problems (Meyer et al., 2006; Bilbo and Schwarz, 2009). Although the link between environmental risk factors, the immune response, and neurological dysfunction is not completely clear at present, accumulating evidence suggests that MIA via infection during pregnancy alters brain development and increases the risk for neurodevelopmental disorders in the offspring (Patterson, 2009, 2011; Malkova et al., 2012; Coiro et al., 2015). In this regard, some studies using animal models have reported a role for the complement system in brain abnormalities in the offspring following MIA.

Using a mouse model of inflammation-induced preterm birth and brain injury, a study has shown that treatment of pregnant mice with lipopolysaccharide (LPS) induced an increase in the levels of C5a in both the amniotic fluid and the fetal brain, as well as cortical abnormalities in the preterm fetuses, characterized by decreased expression of neuronal markers and increased cell death (Pedroni et al., 2014). Interestingly, these fetal cortical brain alterations associated with LPS-induced preterm birth were not observed in fetuses deficient for *C5aR* or born from mice treated with anti-C5 antibody (Pedroni et al., 2014). Furthermore, the neurotoxic effect of C5a was confirmed *in vitro* by treating isolated fetal cortical neurons with this anaphylatoxin, which inhibited the growth of neurites and increased cell death, phenotypes that were blocked by C5aR antagonist (Pedroni et al., 2014). Similar results were observed in another study using a mouse model of malaria in pregnancy, in which the offspring from infected mice has shown impaired learning and memory and depressive-like behavior compared to non-infected controls. The neurocognitive impairments observed in the malaria-exposed offspring were rescued by deletion of *C5aR* in the fetuses or by treating infected pregnant mice with anti-C5 antibody (McDonald et al., 2015). Together, these findings suggest a new role for the anaphylatoxin C5a in the cortical brain damages and behavioral disturbances observed in fetuses exposed to prenatal inflammation.

More recent studies have shown that MIA induced by synthetic dsRNA (polyI:C) in pregnant rodents, which acts to initiate an inflammatory response similar to that caused by viral infection, caused long-term increase in C1q (Han et al., 2017) and *C4* (Duchatel et al., 2018) expression in the cortex of their offspring. The involvement of C1q and C4 in synaptic pruning (Stevens et al., 2007; Schafer et al., 2012; Sekar et al., 2016) suggests that these complement molecules may be involved in MIA-associated brain abnormalities in the offspring, but additional studies are necessary to confirm this possibility.

## Complement System Dysregulation in Neurodevelopmental Disorders

### Complement in Schizophrenia

Schizophrenia (MIM 181500) is a chronic and disabling mental disorder that affects about 1% of the world population (Hafner and der Heiden, 1997; McGrath et al., 2008). The symptoms of schizophrenia include psychosis and deficits in cognition and social interaction, which most commonly emerge in late adolescence or early adulthood (Howes and Murray, 2014). Neuropathological findings in individuals with schizophrenia include abnormal cortical organization possibly due to altered neuronal migration (Akbarian et al., 1996; Arnold et al., 1997; Shenton et al., 2001) and reduced cortical gray matter thickness (Selemon and Goldman-Rakic, 1999; Cannon et al., 2002, 2015) and diminished synaptic density possibly due to excessive pruning of cortical synapses, thus producing hypoconnectivity of prefrontal cortex (Glantz and Lewis, 2000; Glausier and Lewis, 2013). Although the exact mechanisms underlying schizophrenia are not yet fully understood, both complex genetic and environmental risk factors have been implicated in its pathogenesis. Importantly, accumulating evidence suggests that complement dysregulation is among the risk factors for the disorder.

The earliest studies of the complement system in schizophrenia have focused on the complement hemolytic activity to measure both the overall function of the classical pathway and the function of its specific components in blood. Although the results from the different studies were diverse with some studies reporting either a decrease (Spivak et al., 1989, 1993) or no difference (Sasaki et al., 1994) in complement total hemolytic activity in patients compared to control individuals, the majority of data, mainly concerning individual complement components (such as C1, C2, C3, and C4), points toward higher activity of the classical pathway in patients (Maes et al., 1997; Shcherbakova et al., 1999; Hakobyan et al., 2005; Mayilyan et al., 2006; Figure 1). Accordingly, increased levels of C1q attached to circulating immune complexes (C1q-CIC) and increased expression of CR1 on blood cells, which can bind to C1q-CIC and mediate clearance of immune-complexes, were found in patients with schizophrenia (Arakelyan et al., 2011; Figure 1). Also, increased circulating C1q levels were observed in mothers of infants who later developed schizophrenia, which suggest that exposure of the fetal brain to maternally derived C1q might be a contributory factor for the disease (Severance et al., 2014), as suggested by rodent models of MIA. In addition, it is noteworthy that decreased expression of *CSMD1*, which codes for an inhibitor of the classical complement pathway, was observed in serum from patients with schizophrenia (Liu et al., 2019).

Curiously, a negative correlation was recently found between superior frontal cortical thickness and the expression levels of *C5* and *SERPING1* genes in peripheral blood mononuclear cells from a sample of adult Swedish twins enriched for schizophrenia patients, who are known to show reduced cortical gray matter thickness (Allswede et al., 2018). Whereas C5 is an activator of the complement system, *SERPING1* codes for a protease involved in the inhibition of the classical and lectin complement cascades, and its increased expression could reflect a compensatory mechanism for higher complement activity. Although this finding cannot establish causality and further studies are clearly needed, it is tempting to speculate that the relationship between enhanced expression of these specific complement components in the blood and cortical thickness may represent a trace of earlier immune dysregulation during development.

While several lines of evidence support the involvement of the classical complement pathway in schizophrenia susceptibility, only a few studies have addressed specifically the role of the lectin and alternative pathways in the disease. Data on the involvement of the lectin cascade show higher MBL-bound MASP2 activity in serum from individuals with schizophrenia, suggesting increased activity of this pathway in the disorder (Mayilyan et al., 2006; Figure 1). On the other hand, conflicting results exist regarding the involvement of the alternative cascade, because a hemolysis-based assay indicated an upregulation of the alternative pathway (Boyajyan et al., 2010), while an ELISA-based assay identified suppression of the same cascade in the peripheral blood from patients with schizophrenia (Li et al., 2012). Therefore, further studies are required to confirm whether dysfunction of lectin and alternative complement cascades contribute somehow to schizophrenia.

As mentioned above, all of the complement pathways converge at the point of C3 and data related to C3 levels in blood from schizophrenia patients are also controversial. Some studies found no alteration (Spivak et al., 1993) or increased levels of C3 in patients compared to controls (Maes et al., 1997; Shcherbakova et al., 1999; Hakobyan et al., 2005; Boyajyan et al., 2010), while others found a decrease and suggest a negative correlation between the serum levels of C3 and the severity of the symptoms (Wong et al., 1996; Li et al., 2016; Figure 1). The reasons for these differences are unknown, but may include either population-specific genetics and environmental risk factors or the stage of illness (acute vs. chronic). Thus, additional studies using larger groups are needed to elucidate the precise nature of the relationship between C3 levels and schizophrenia susceptibility.

Genetic studies evaluating the association between complement gene polymorphisms and schizophrenia have also been conducted. Initial studies have yielded unreliable or conflicting results due to lack of replication or small sample sizes (Rudduck et al., 1985; Fananas et al., 1992; Wang et al., 1992; Schroers et al., 1997; Mayilyan et al., 2008; Zakharyan et al., 2011). However, more recent large-scale genome-wide association studies evaluating very large sample collections for hundreds of thousands of single-nucleotide polymorphisms (SNPs) have shown variants significantly associated with higher risk of schizophrenia at *CSMD1* (Schizophrenia Psychiatric Genome-Wide Association Study (GWAS) Consortium, 2011) and *C4* genes (Sekar et al., 2016). *CSMD1* is highly expressed in the CNS (Kraus et al., 2006) and its schizophrenia risk allele was associated with poor performance on neuropsychological measures of general cognitive ability and memory function (Donohoe et al., 2013; Koiliari et al., 2014). C4 is encoded by two different genes, *C4A* and *C4B*, which vary in structure and copy number leading to a wide range of expression levels of each isotype. Interestingly, the strongest association with schizophrenia was found with alleles that increase expression of *C4A* (Sekar et al., 2016). Accordingly, higher *C4A* expression was observed in brain samples from patients with schizophrenia compared to controls (Sekar et al., 2016) and a positive correlation was found between the copy numbers of *C4* and neuropil contraction in different brain regions in patients (Prasad et al., 2018; Figure 1), which strongly suggest that increased C4 levels constitute a risk factor for schizophrenia.

Whereas the abnormal complement expression in the peripheral blood of both patients with schizophrenia and their mothers suggests that complement dysfunction as part of standard immune pathways may contribute to schizophrenia in a subset of patients, findings from several of the abovementioned studies also suggest that the clinical and neuropathological phenotypes of schizophrenia may also be, at least in part, due to dysfunction of locally synthesized complement in the brain during specific periods of neural development. The strong association between increased *C4A* expression with schizophrenia (Sekar et al., 2016), the involvement of classical complement cascade in synapse elimination (Stevens et al., 2007; Schafer et al., 2012; Bialas and Stevens, 2013; Sekar et al., 2016; Comer et al., 2019), and the decreased brain connectivity and sociability in mice overexpressing *C4* in the prefrontal cortex (Comer et al., 2019) strongly suggest that enhanced complement-mediated synaptic pruning contributes directly to reduction in cortical gray matter thickness and to schizophrenia pathogenesis. On the other hand, while it is highly attractive to speculate that complement-mediated dysregulation in neuronal migration may contribute to the pathogenesis of schizophrenia, further detailed studies are still required to directly establish a causal link.

### Complement in Autism Spectrum Disorder and in Rett Syndrome

Autism spectrum disorder (MIM 209850) comprises a heterogeneous group of early onset neurodevelopmental diseases characterized by impairments in social-communicative skills and repetitive behaviors (APA, 2013) that affects at least 1.5% of the population worldwide (Christensen et al., 2016). The most consistent neuropathological findings in patients with ASD include increased cortical surface area during early childhood (Miles et al., 2000; Hazlett et al., 2011) and reduced number of Purkinje cells in the cerebellum (Allen, 2005) possibly due to abnormal progenitor cell neurogenesis, altered cortical organization (presence of heterotopias and more frequent and narrower minicolumns) suggestive of abnormal neuronal migration (Casanova et al., 2002; Whitney et al., 2008; Stoner et al., 2014), and increased cortical dendritic spine densities possibly due to defective synapse elimination during brain development (Hutsler and Zhang, 2010; Tang et al., 2014). Recent molecular genetic studies have identified a specific cause for ASD in almost 30% of the cases, while in the remaining cases the underlying pathogenic mechanisms may involve complex genetic and environmental risk factors (Bourgeron, 2015). Preliminary evidence suggests a possible role for the complement system in the pathogenesis of ASD.

Genetic association studies have reported that the complement *C4B* gene null allele has increased frequency in patients with ASD compared to control individuals (Warren et al., 1991; Odell et al., 2005; Mostafa and Shehab, 2010) and, accordingly, a significant decrease in the plasma levels of C4B protein was observed in ASD patients (Warren et al., 1994; Figure 1). On the other hand, proteomic analyses have suggested that the levels of other complement system proteins, such as C1q, C3, and C5, are elevated in plasma from patients with ASD (Corbett et al., 2007; Shen et al., 2018; Figure 1). In addition, a significantly increased activity of CFI, a negative regulatory component of the alternative pathway responsible for cleavage and inactivation of C3b and C4b, has been observed in plasma from ASD patients (Momeni et al., 2012).

The expression of complement system components in brain tissues from patients with ASD was also investigated and some conflicting results were obtained. While a genome-wide DNA methylation profiling of prefrontal cortex (Brodmann areas BA10 and BA24) has shown hypomethylation and, consequently, overexpression of *C1q*, *C3*, and *CR3* genes in the ASD brains (Nardone et al., 2014), a more recent study found decreased mRNA levels of *C1q*, *C3*, and *C4*, and increased mRNA levels of *C2*, *C5*, and *MASP1* in the middle frontal gyrus from patients compared to controls (Fagan et al., 2017; Figure 1). The discrepancies obtained in the expression of *C1q* and *C3* genes in brain tissues from patients with ASD could be due to the analysis of different brain regions and/or analysis of different subgroups of patients combined with small sample sizes and need to be clarified in further studies.

Collectively, the evidence regarding association between complement dysfunction and ASD is far weaker than the evidence of complement dysregulation in schizophrenia susceptibility, and a recent large-scale genome-wide association study did not report common variants in complement genes significantly associated with ASD (Grove et al., 2019). However, the altered complement system expression in peripheral blood and in brain from patients with ASD might suggest that an aberrant activity of this system may somehow contribute to ASD. Based on decreased expression of some complement components in post-mortem brain of ASD patients (Fagan et al., 2017), the increased number of dendritic spines and glutamate synapses in ASD brains (Hutsler and Zhang, 2010; Tang et al., 2014) as well as in *C1q-* and *C3*-deficient mouse brains (Chu et al., 2010; Perez-Alcazar et al., 2014), the reduced elimination of retinogeniculate synapses in mice lacking *C4* (Sekar et al., 2016) and the ASD-like phenotypes observed in *C3-*deficient mice (Perez-Alcazar et al., 2014; Fagan et al., 2017), it is tempting to speculate that diminished complement-mediated synaptic pruning, among other known mechanisms (Tang et al., 2014), may contribute to the cortical hyperconnectivity and behavioral phenotypes in ASD. Nevertheless, additional studies are clearly necessary to further explore the possible role of complement dysregulation in key aspects of ASD neuropathology.

Finally, it is noteworthy that, as occurs with schizophrenia and ASD, immune dysregulation may also contribute to some of the neuropathological features of Rett syndrome (RTT; MIM 312750) (Cortelazzo et al., 2014; De Felice et al., 2014; O’Driscoll et al., 2015), an X-linked progressive neurodevelopmental disorder primarily affecting girls at a frequency of 1:10,000 live female births. Although RTT is not classified as an ASD and is recognized as a distinct pathological entity (APA, 2013) (DSM-5), RTT may show overlapping symptoms with ASD, and is characterized by typical early development until age 6–18 months followed by a rapid deceleration in growth associated with progressive loss of acquired motor and language skills, severe cognitive impairment, intractable seizures, spasticity and stereotypic hand movements (Chahrour and Zoghbi, 2007). More than 95% of classic RTT cases are caused by sporadic loss-of-function mutations in the gene encoding MECP2 (Amir et al., 1999), a transcriptional regulator of gene expression that acts through epigenetic mechanisms on chromatin structure (Nan et al., 1998; Bienvenu and Chelly, 2006). Interestingly, a proteomic study has shown that the levels of several proteins involved in the immune system are altered in plasma from patients with RTT, including overexpression of complement factor B of the alternative complement pathway (Cortelazzo et al., 2014). Also, a recent genome-wide transcriptome (RNA-seq) analysis of post-mortem brain samples (from both frontal and temporal cortex) from young RTT patients showed that all three genes encoding complement C1q complex (*C1QA*, *C1QB*, *C1QC*) were downregulated in RTT human brains as they are in *Mecp2* knockout mice (Lin et al., 2016), which suggest that the expression of these genes is regulated by MECP2. Although the involvement of C1q in RTT pathogenesis has not yet been clarified and additional studies are required, the role of C1q in regulating dendritic spine density (Chu et al., 2010; Perez-Alcazar et al., 2014) may contribute somehow to RTT.

### Complement in 3MC Syndrome

3MC syndrome (MIM 257920; 265050; 248340) comprises a rare developmental disorder that unifies four autosomal recessive diseases with overlapping features previously known as Mingarelli, Malpeuch, Michels, and Carnevale syndromes (Titomanlio et al., 2005). This syndrome is characterized by a spectrum of developmental anomalies that include facial dysmorphism, cleft lip and/or palate, postnatal growth deficiency, learning disability and hearing impairment. Less often, individuals with 3MC syndrome may also show craniosynostosis, genital, limb, and vesicorenal anomalies (Titomanlio et al., 2005; Leal et al., 2008).

Several different potentially deleterious mutations in genes of the lectin arm of the complement system - such as *MASP1*, *COLEC10*, and *COLEC11* genes – have been described in patients with 3MC syndrome (Sirmaci et al., 2010; Rooryck et al., 2011; Atik et al., 2015; Urquhart et al., 2016; Gardner et al., 2017; Munye et al., 2017; Graul-Neumann et al., 2018; Basdemirci et al., 2019). It has recently been shown that disease-associated mutations in *COLEC10* and *COLEC11* inhibit either production or secretion of encoded proteins in mammalian cells (Rooryck et al., 2011; Venkatraman Girija et al., 2015), which may explain the undetectable levels of CL-K1 in the serum of affected individuals (Rooryck et al., 2011). However, it is noteworthy that although at least some of these mutations impair the normal function of the encoded proteins, normal levels of downstream reaction cascade components (C2, C3, and C4) were found in serum from patients, suggesting a possible direct role of these lectin cascade proteins in 3MC syndrome pathogenesis independently of standard lectin pathway activation (Rooryck et al., 2011).

Studies using zebrafish (Rooryck et al., 2011) and an *in vitro* cell model (Munye et al., 2017) have shown that these components of the lectin pathway act as chemoattractants to guide cell migration and suggest that the craniofacial abnormalities in 3MC syndrome are the result of deficient migration of neural crest cells during development (Rooryck et al., 2011). Also, the involvement of components of the lectin pathway in regulating neuronal migration in the developing cerebral cortex (Gorelik et al., 2017a) also suggests that the cognitive impairment observed in 3MC patients may be explained in part by deficits in neuronal migration. Further studies, however, are necessary to further understand the mechanisms by which dysfunctional *MASP1*, *COLEC10*, and *COLEC11* genes may lead to 3MC syndrome.

## Concluding Remarks

Here, we have highlighted the emerging functions of the complement system as a key regulator of normal CNS development. Components of lectin and terminal complement pathways, mainly the C3a and C5a anaphylatoxins, have been shown to regulate neural progenitor cell proliferation, neurogenesis and neuronal migration. Components of classical and terminal complement pathways, such as C1q, C3, and C4, have been shown to tag weaker synapses for removal by microglia, which sculpts brain connectivity. Consequently, alterations in the expression of complement components in the brain may lead to long-lasting changes in brain development and function. The severe autosomal recessive 3MC syndrome originates from rare pathogenic mutations in genes of the lectin pathway that regulate cell migration during embryonic development. In both schizophrenia and ASD, evidence is growing that abnormal complement signaling owing to genetic mutations or as a result of inflammatory insults during prenatal or early postnatal development may lead to changes in brain connectivity and may contribute to disease pathophysiology. Although research into the molecular mechanisms downstream from complement components has just begun to be explored, the progress in this field holds tremendous promise not only for increasing our understanding of neurodevelopment, but also for elucidating and potentially even treating neurodevelopmental disorders.

## Author Contributions

All authors have made a substantial, direct and intellectual contribution to the work, and approved it for publication.

## Conflict of Interest

The authors declare that the research was conducted in the absence of any commercial or financial relationships that could be construed as a potential conflict of interest.
